# Supplementary figures and images for: Neural signaling contributes to heart formation and growth in the invertebrate chordate, Ciona robusta
Source: PLoS Biol. 2026 Apr 14;24(4):e3003715. doi: 10.1371/journal.pbio.3003715 (PMC13078634; doi:10.1371/journal.pbio.3003715)

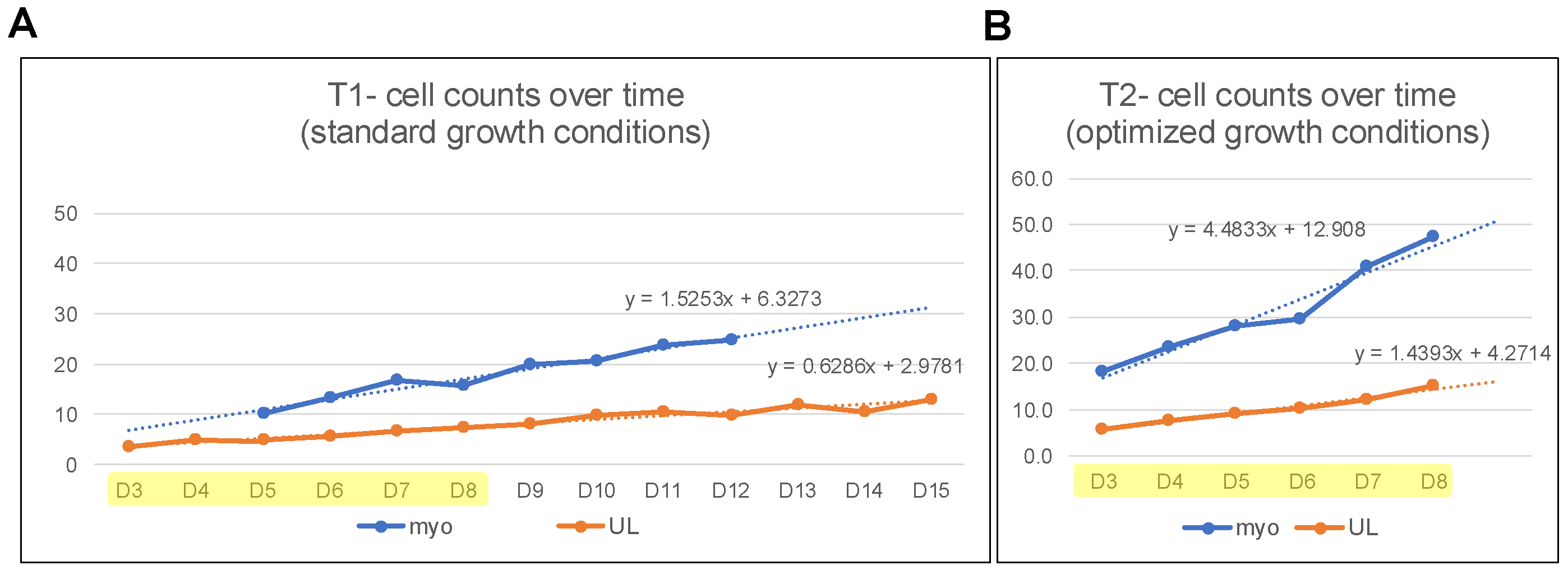

Supplement: S1 Fig — (A) Quantity of myocyte (blue) and midline UL cells (orange) from D3 to D15 using standard culturing conditions. (B) Quantity of myocyte (blue) and midline UL cells (orange) from D3 to D8 days using optimized culturing conditions (see Methods section). In A and B, D3-D8 X-axis labels are highlighted for comparison. Orange dots and lines, number of UL cells per time point. (TIF) [file pbio.3003715.s001.tif]

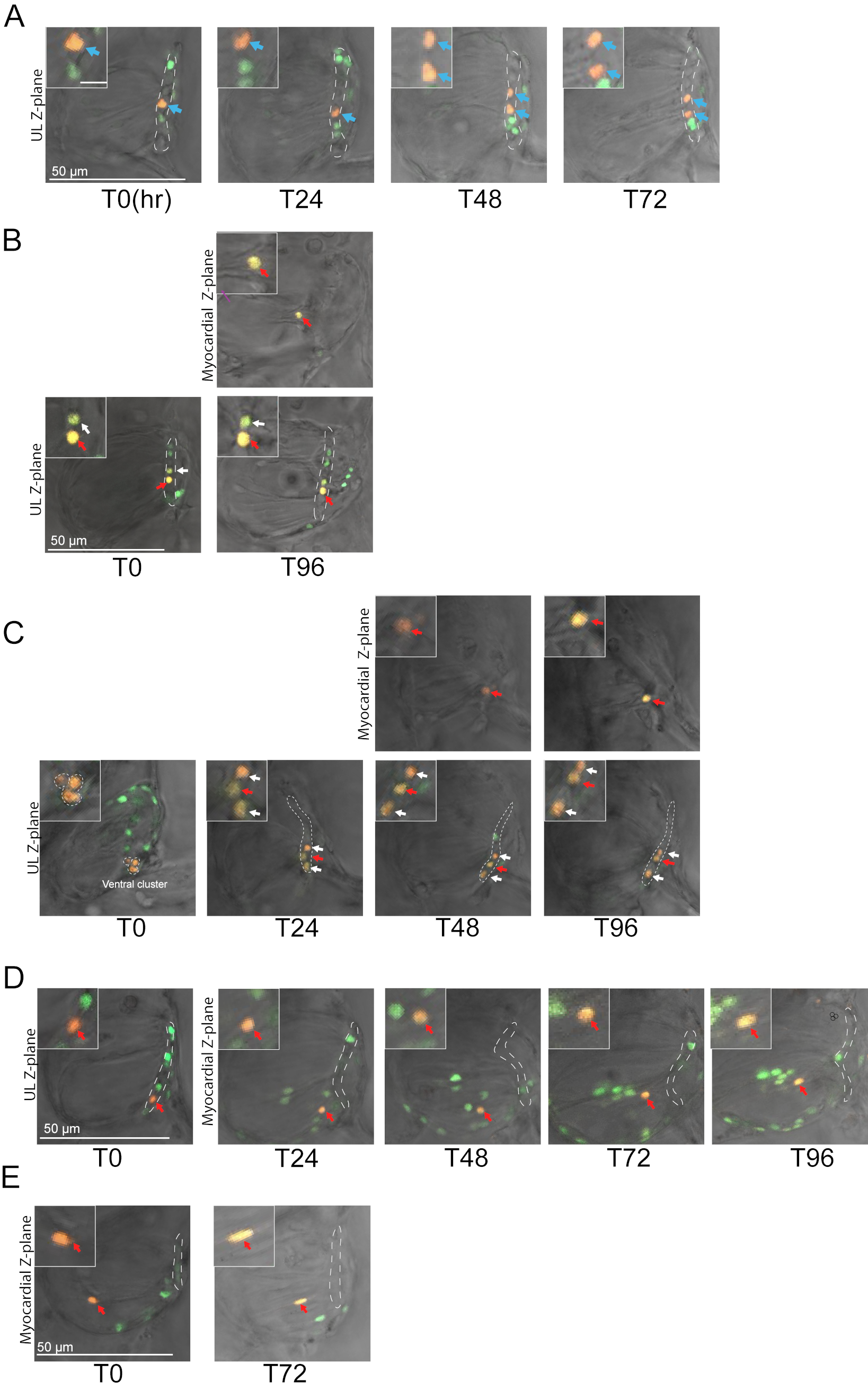

Supplement: S2 Fig — In all panels, photoconverted cells (yellow) are tracked over multiple days in living juveniles starting with photoconversion at D5, timepoints indicate hours after conversion. See text for details. (A) Blue arrows track a single labeled midline UL cell that appears to divide symmetrically to produce two midline UL daughters. (B, C) Red arrows track single labeled midline UL cells that appears to divide asymmetrically to produce a midline UL daughter that remains in the same confocal plane as the rest of the UL, bottom row, along with a presumptive myocardial precursor that moves into a different confocal plane (top row). White arrows indicate neighboring cells in the UL. (D) Red arrows track a single labeled presumptive myocardial precursor that appears to migrate anteriorly, away from the UL. (E) Red arrows track a single labeled presumptive myocardial precursor that appears to mature over a 72-hour time-course as evidenced by elongation of the nucleus. (TIF) [file pbio.3003715.s002.tif]

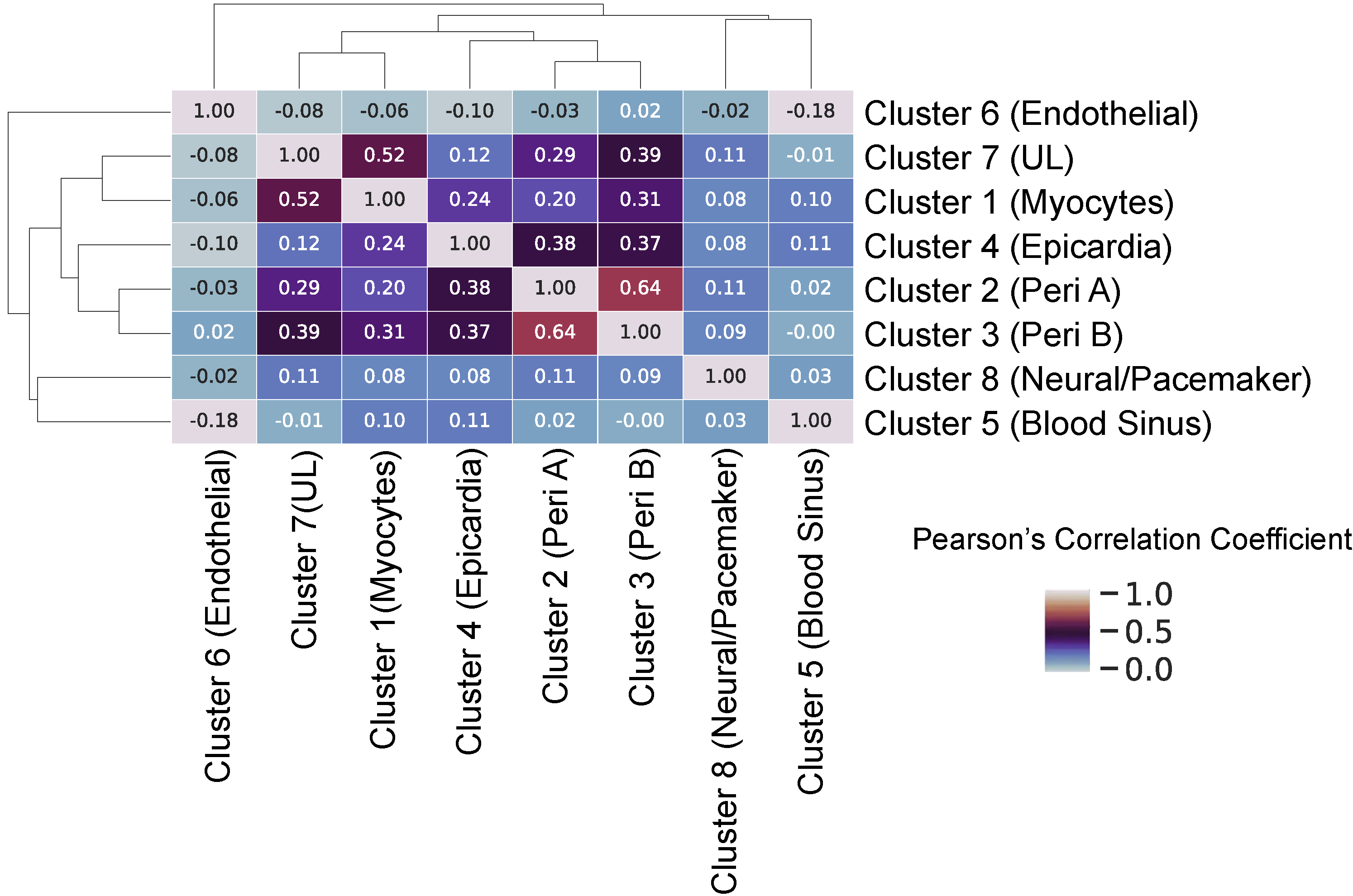

Supplement: S3 Fig — PCC suggests Cluster 2 correlates strongly with Cluster 3 and that Cluster 1 correlates strongly with Cluster 7. Overlapping expression patterns between Clusters 1 and 7 may contribute to the lack of uniquely enriched genes in Cluster 1 (see main text and Fig 3A and 3B). Additionally, PCC revealed Clusters 5 and 8 did not correlate strongly with any other clusters suggesting these clusters are transcriptionally unique. (TIF) [file pbio.3003715.s003.tif]

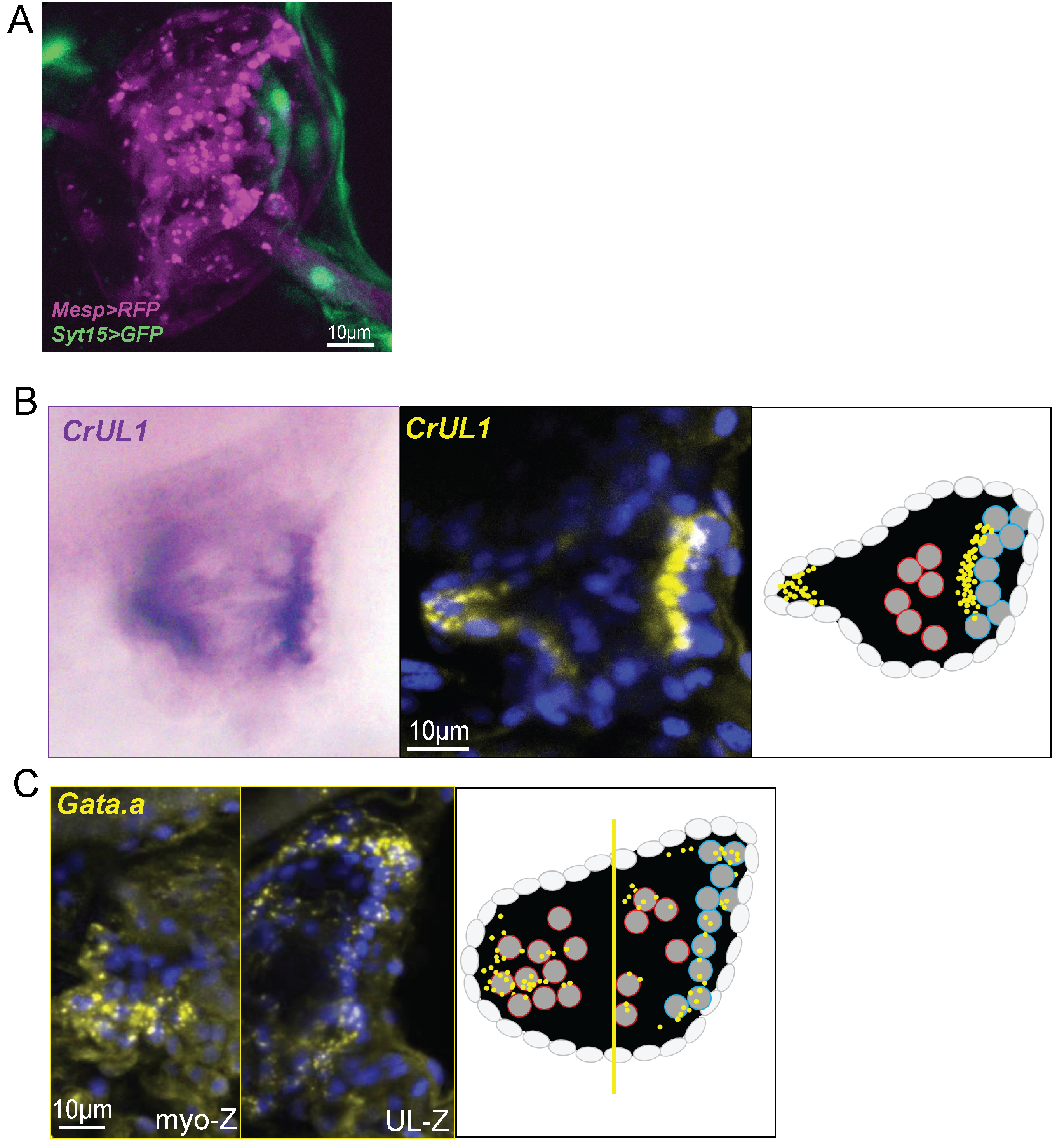

Supplement: S4 Fig — (A) Representative image displaying Synaptotagmin 15 reporter (Syt15>GFP) expression in a D15 juvenile. Mesp>RFP labels pericardium, myocardium, and the UL (magenta). Syt15>GFP (green) was detected in the overlying epicardium along with a few large underlying cells which may be neural-like cells matching scRNA-seq data indicating that Synaptotagmin 15 is expressed in the presumed epicardial and neural-like clusters (#4 and #8, Fig 3). (B, C) In situ expression patterns of marker genes associated with the presumptive UL cluster (#7, Fig 3). (B) CrUL1 expression in a D5 heart. Colorimetric in situ hybridization (left), FISH (middle), and cartoon schematic (right). (C) Gata.a expression in a D8 heart. FISH (left) and cartoon (right). Note that in the micrograph a Z-plane containing myocardial cells in the anterior region of the heart is shown on the left while the Z-plane containing the UL in the posterior region of the heart is shown on the right. Blue (B, C) represents DAPI staining. Yellow (B, C) represents probe detection for each transcript. All images shown anterior to the left and dorsal up. (TIF) [file pbio.3003715.s004.tif]

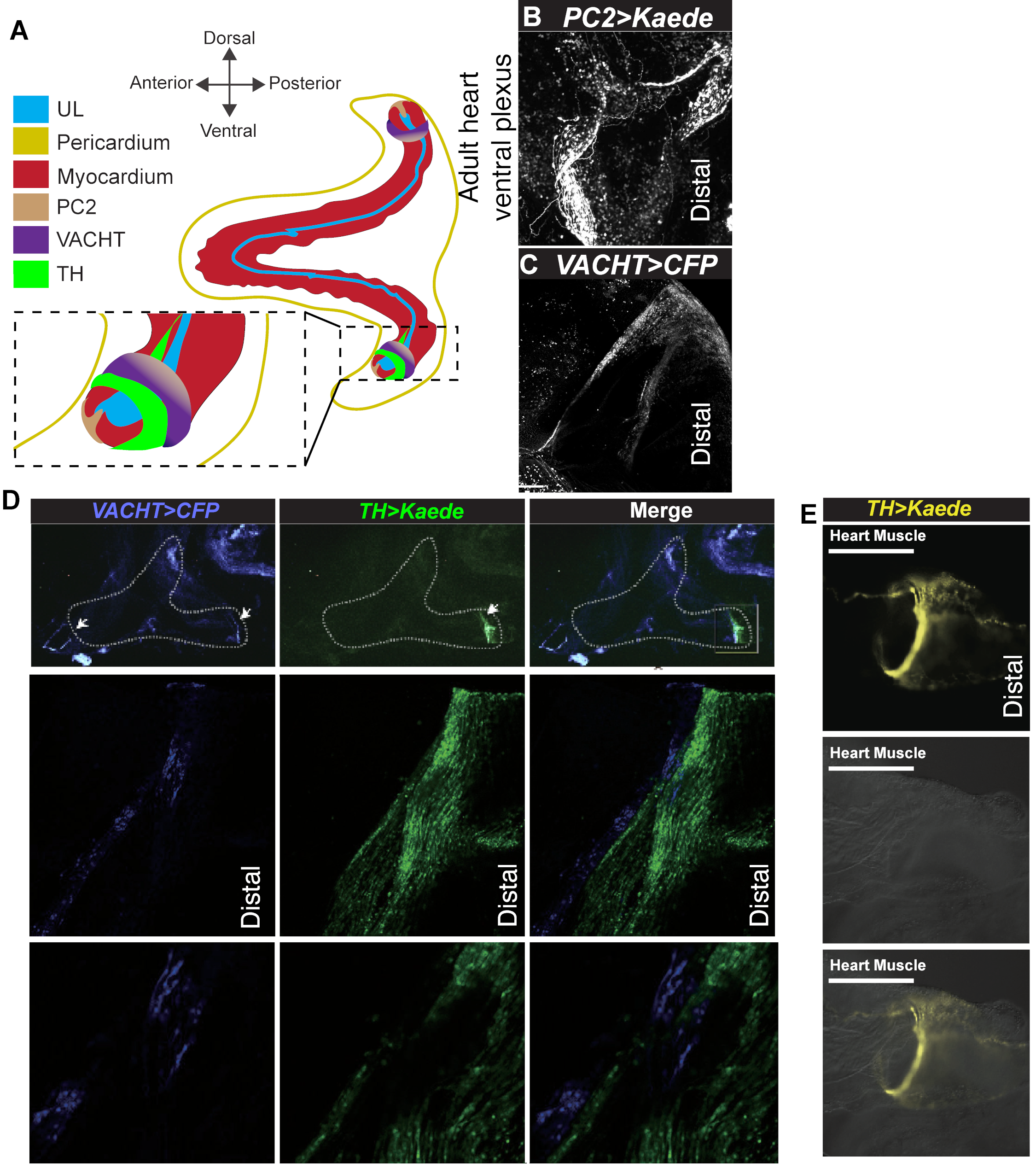

Supplement: S5 Fig — (A) Summary diagram of observed localization patterns for PC2+, VACHT+, and TH+ neural-like cells in the distal plexus of the adult heart. Note the ventral-exclusive presence of TH (green). (B) PC2>Kaede expression. (C) VACHT>CFP expression. (D) VACHT>CFP and TH>Kaede double-labeled adult hearts. Left column is VACHT>CFP (blue), middle column is TH>Kaede (green), and right column is merged. Middle and bottom rows correspond to enlarged areas of two different Z-planes of the boxed area in the top row. (E) TH>Kaede reporter expression at the distal end of the ventral plexus. Top panel is Kaede fluorescence, middle panel is brightfield, bottom panel is merged. Line in E indicates where the myocardial tube ends relative to the TH+ neural-like ring. (TIF) [file pbio.3003715.s005.tif]

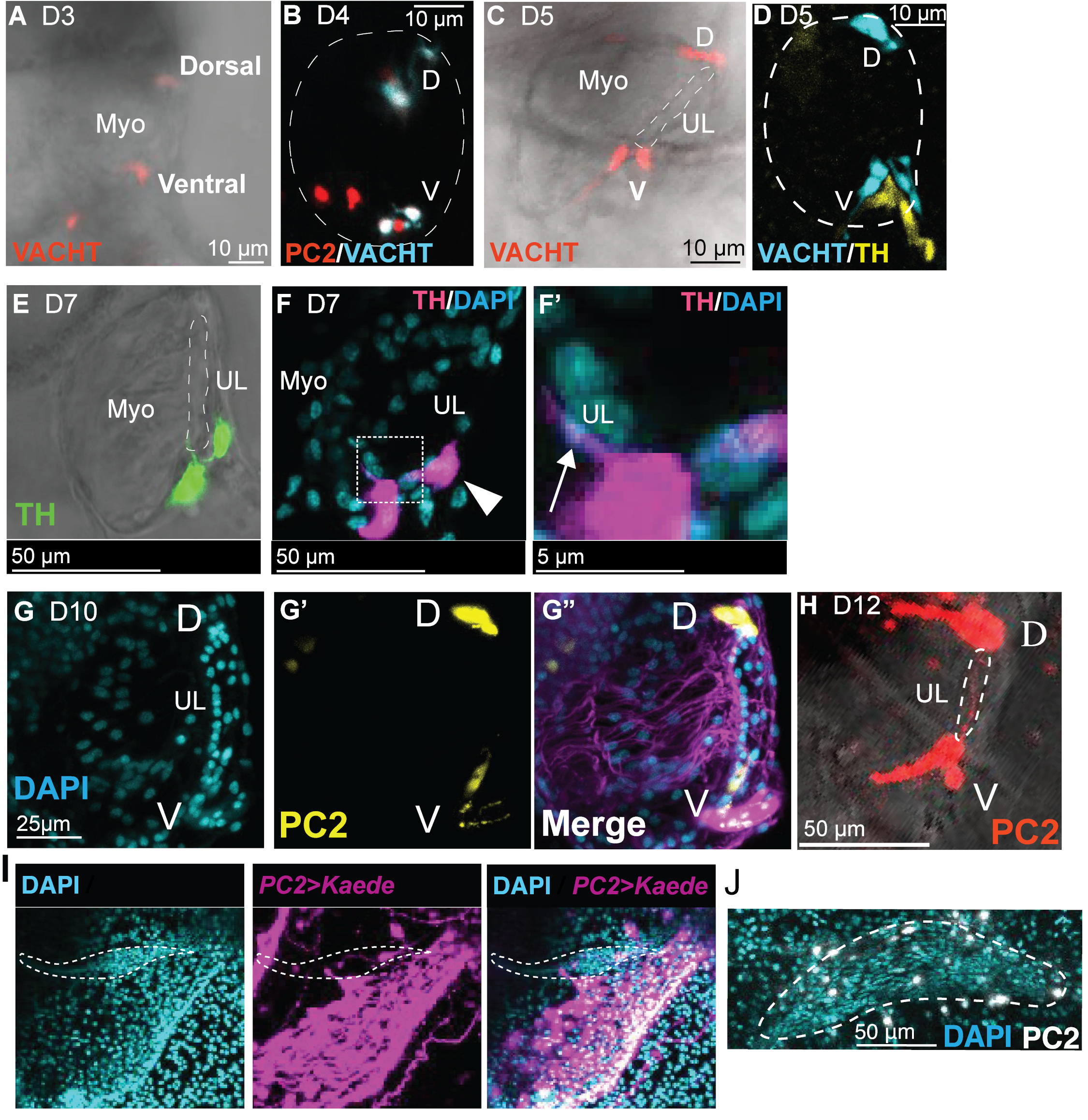

Supplement: S6 Fig — (A) VACHT>CFP (red) in live D3 juvenile heart. (B) PC2>Kaede (red) and VACHT>CFP (blue) in a fixed D4 juvenile heart, outlined in white. (C) VACHT>CFP (red) in live D5 juvenile heart. (D) VACHT>CFP (blue) and TH>Kaede (yellow) in a fixed D5 juvenile heart, outlined in white. (E) TH>Kaede in a live D7 juvenile heart. (F) TH>Kaede (magenta) in a fixed juvenile heart. DAPI staining in cyan. F′ shows a single Z-plane from F, note the overlap in magenta and cyan that appears to be associated with a UL cell (arrow). (G) DAPI stained nuclei of a D10 transgenic PC2>Kaede juvenile heart. G′ shows PC2>Kaede expression in the same heart. G″ shows the merged view. Note the overlap between PC2> Kaede expression and the distal UL clusters which is particularly clear at the dorsal end of the UL. (H) PC2>Kaede expression (red) in a D12 juvenile heart. Note staining at the dorsal and ventral ends of the UL as well as expression along the UL (within the dotted line). (I, J) PC2>Kaede labels cells that are interspersed with densely clustered DAPI-stained nuclei within the distal UL (outlined in white) in adult hearts. In both panels, the distal UL is distinguished by densely packed nuclei and is outlined by a dotted white line. PC2>Kaede is displayed in magenta (I) or white (J). A, C, E, and H, fluorescence merged with brightfield. In these images the UL is often outlined by a white dotted line and Myo indicates the position of the myocardial tube. (TIF) [file pbio.3003715.s006.tif]

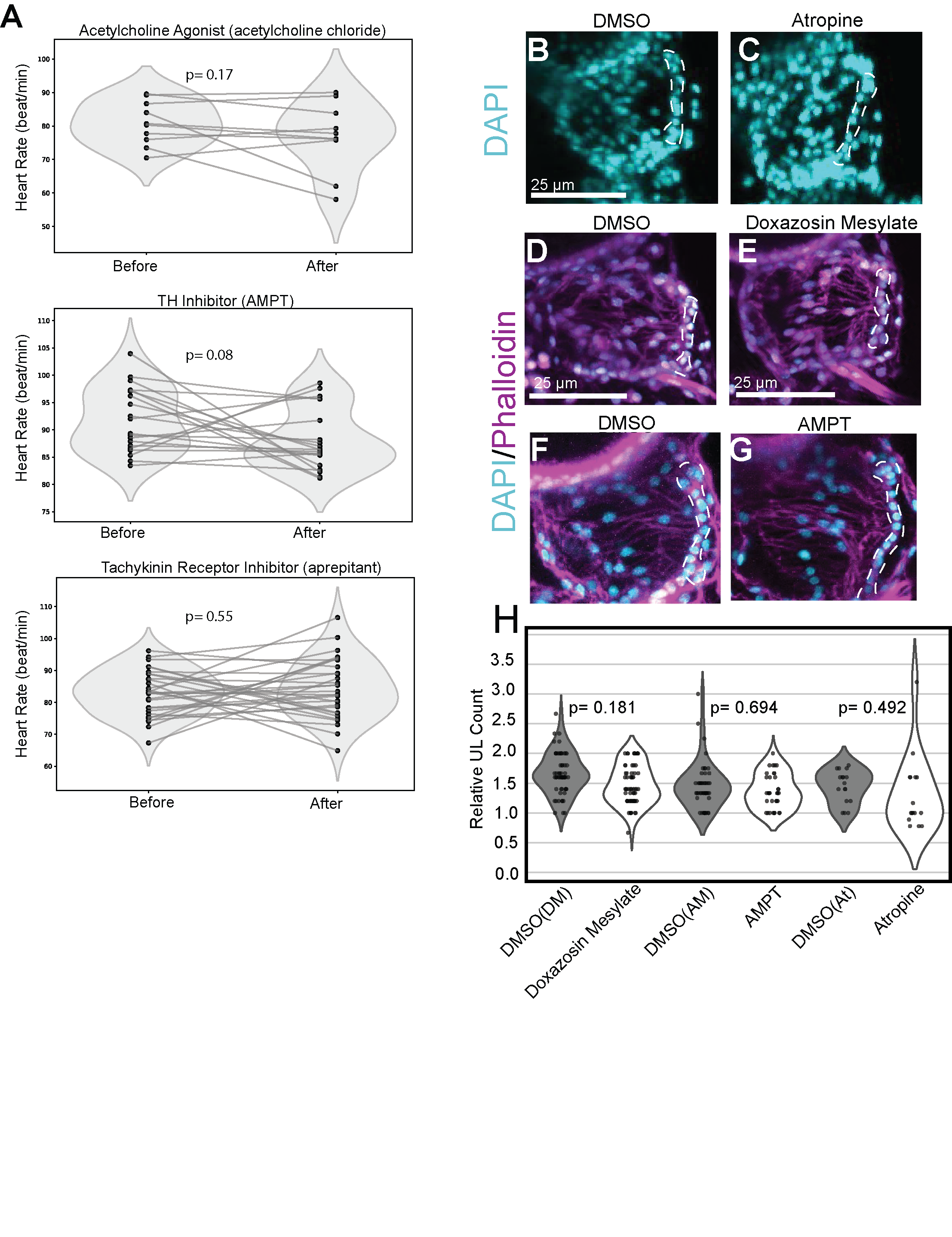

Supplement: S7 Fig — (A) Violin plots of recorded heart rates in response to acetylcholine chloride (top), AMPT (middle), and Aprepitant (bottom). (B) D7 control heart. (C) D7 heart treated with Atropine. (D) D7 control heart. (E) D7 heart treated with Doxazosin Mesylate. (F) D7 control heart. (G) D7 heart treated with AMPT. (H) Violin plot of normalized UL counts per treatment. Counts normalized to the DMSO control for each trial. In B–G, cyan represents DAPI-stained nuclei. Images in D–G also show phalloidin staining (magenta). For H, a t test was performed on normalized data, averaged across trials. Doxazosin Mesylate: N = 60 control and 58 experimental samples, 2 trials. Atropine: N = 13 control and 14 experimental samples, 2 trials. AMPT: N = 46 control, 33 experimental samples, 2 trials. The data underlying the graphs in this Figure can be found in S1 Data. (TIF) [file pbio.3003715.s007.tif]

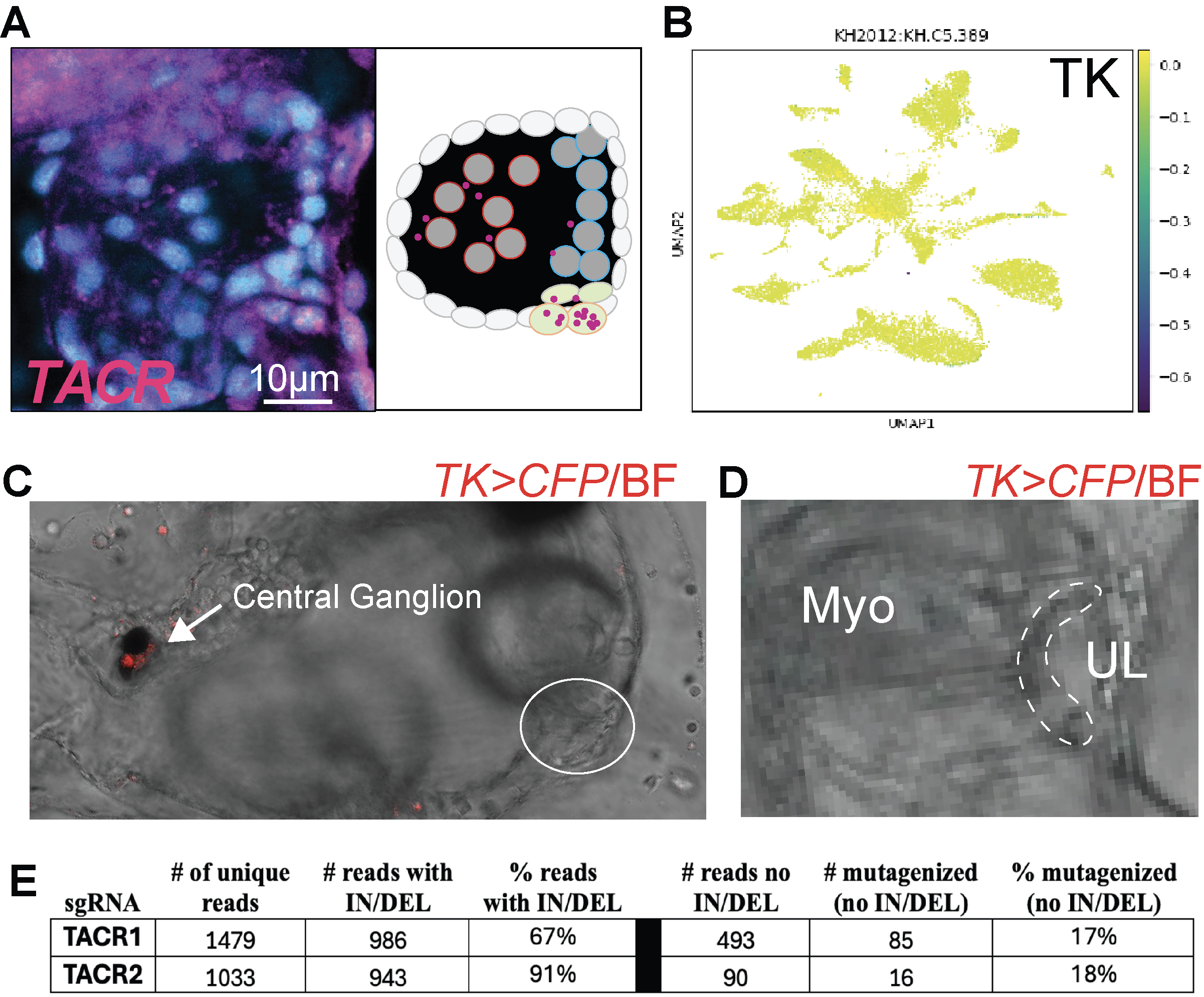

Supplement: S8 Fig — (A) Fluorescent in situ hybridization of TACR expression. Blue represents DAPI and magenta displays fluorescent probe detection (left). Cartoon model of expression pattern (right), UL cells outlined in blue, myocardial cells outlined in red, green cells represent presumptive neural-like/pacemaker cells. (B) UMAP showing TK expression levels, note absence of expression. (C) Fluorescent TK>CFP reporter electroporation expression in the central ganglion (red). (D) Magnified region of the heart. Note absence of reporter expression, UL outlined in white, Myo indicates position of the myocardium. (E) Table displaying data generated from transgenic larvae co-electroporated with Ef1a>Cas9 along with either TACRsgRNA1 or TACRsgRNA2 and then subjected to Genewiz SNP/Indel analysis. For each sample, ~ 1,000–1,500 unique reads were analyzed for the presence of naturally occurring IN/DELs and for mutagenesis within the targeted locus. This analysis revealed a highly prevalent naturally occurring IN/DEL near both targeted loci (67% for TACRsgRNA1 and 91% for TACRsgRNA2, column three). In these reads, there was a high level of sequence variation in the targeted locus (S3 Table). Thus, we manually filtered through the unique reads generated by this analysis for each sgRNA (TACRsgRNA1and TACRsgRNA2) to distinguish whether or not they contained the prevalent IN/DEL. We then assessed the incidence of mutagenesis within the unique reads that did not contain the IN/DEL (17% for TACRsgRNA1 and 18% for TACRsgRNA2, column six). These percentages align with the penetrance observed when both sgRNAs were used for CRISPR (Fig 7). We suspect that the high incidence of the IN/DEL may reflect a change in the C. robusta population used for this follow-up assay. This sequencing assay was conducted using gametes from adults harvested from a Los Angeles population that is distinct from population used in our CRISPR experiments. As expected for targeted mutagenesis, all detected deletions that potenti [file pbio.3003715.s008.tif]
