# Supplementary material for: Neural signaling contributes to heart formation and growth in the invertebrate chordate, Ciona robusta
Source: PLoS Biol. 2026 Apr 14;24(4):e3003715. doi: 10.1371/journal.pbio.3003715 (PMC13078634; doi:10.1371/journal.pbio.3003715)
Supplement: Attachment — Submitted filename: Response to Reviewers V2.docx [file pbio.3003715.s021.docx]

Dear Dr. Hart,

Thank you for your editorial suggestions and for your patience as we have worked to revise and resubmit this manuscript.

We greatly appreciate the thoughtful comments provided by the reviewers and their recognition regarding the significance of our findings and the strength of our data. As stated by Reviewer 1 “The authors used robust experimental methods. Their findings are compelling and well-presented.” Similarly, Reviewer 3 writes “The paper by Gruner et al. provides novel insights into how neural signals are involved in the development of heart cells during Ciona robusta development. The manuscript is well written, and the experiments are comprehensive and clearly described. The results are thoroughly discussed and are of significant interest to the community.” Their support for the paper is mirrored by the subsequent publication of a perspective highlighting the importance of this work in the journal Development ([link](https://journals.biologists.com/dev/article/152/15/dev205088/368789/In-preprints-it-takes-nerves-to-grow-a-heart)).

We also appreciated the concerns raised by Reviewers 1 and 2 and have worked to thoroughly address them. As pointed out by Reviewer 2, the paper contains an unwieldy amount of data, much of which was not directly relevant to the main conclusions of the paper and was thus covered by numerous supplemental figures. Both of these reviewers also expressed concerns regarding the clarity of our presentation or interpretation of some of these supplementary data sets. To address these concerns, we have trimmed out some of these derivative, less-well supported data sets from the paper, greatly reducing the total number of supplementary figures. By narrowing the scope of the paper, we were able to more effectively focus on the most critical findings. Reviewer 2 also stated that “They next show that pharmacological inhibition of tachykinin peptide signalling reduces the number of progenitors. This is somewhat supported by knockdown of tachykinin or its receptor using CRISPR-Cas9 approaches, which are mosaic and lead to a variable phenotype with partial penetrance. In some cases, the formation of the myocardial tube is affected.” This concern was also highlighted by the editorial team. To clarify why CRISPR-Cas9 knockdown leads to mosaic, partially penetrant phenotypes we have cited numerous instances from the literature in which this technique yielded similar variable results in *Ciona robusta* (our model organism). We also conducted a high throughput sequencing assay to directly demonstrate the partial penetrance of CRISPR mediated mutagenesis for one of our targeted loci. To provide additional strong support for this hypothesis, we have also conducted a pharmacological gain of function assay and the results suggest that stabilizing tachykinin signaling is sufficient to increase progenitor proliferation. **Thus, we feel we have thoroughly addressed this key concern, providing substantive support for our central hypothesis (that tachykinin signaling contributes to progenitor proliferation) using two different pharmacological inhibitors, two distinct targeted CRISPR assays along with a pharmacological gain of function assay.** We have also made sure to thoroughly address each of the minor concerns regarding formatting, typos or the clarity of our writing that were raised by the reviewers. Below you will find our detailed response to each individual review.

REVIEWS:

Reviewer #1: The manuscript titled "Neural signaling contributes to heart formation and growth in the invertebrate chordate, Ciona robusta" by H.N Gruner and colleagues represents a thorough investigation of the heart formation in post-metamorphosis Ciona, an invertebrate chordate belonging to the closest living relative to vertebrates. Its study will enhance our understanding of the vertebrate heart's evolution and potentially uncover new therapeutic avenues for heart regeneration. The authors used a combination of lineage tracing by both photoconversion and EdU pulse-chase labeling, single-RNA seq, reporter assays, in situ hybridization, chemical inhibition, and CRISPR/Cas9 mutagenesis to decipher the role of a structure named "undifferentiated line (UL)," Wnt signaling and innervation in Ciona heart formation. They found that cardiomyocyte progenitors are localized in the UL in juveniles. These progenitors first divide asymmetrically in its distal part, one cell staying in this cluster while the other moves toward the midline of the UL, where they can divide either symmetrically or asymmetrically, leaving the midline to differentiate into cardiomyocytes. The authors then performed single-cell RNA sequencing of the adult heart and identified clusters corresponding to cell types composing the heart, including its innervation. They followed up these assays by analyzing the role of Wnt signaling in heart formation, finding that its inhibition increases UL proliferation while its overactivation inhibits UL proliferation. They then characterized the intrinsic and extrinsic innervation of the heart as well as the pacemaker cells of the heart. They identified both cholinergic and dopaminergic intrinsic neurons. However, they do not seem to affect heart rate. They then studied the role of tachykinin since some of these neurons express tachykinin receptors. They discovered that tachykinin promotes the proliferation of UL distal clusters using receptor antagonists and mutagenesis of the tachykinin gene in the cerebral ganglion and the tachykinin receptor gene in the heart lineage.

The authors used robust experimental methods. Their findings are compelling and well-presented. The manuscript is also easy to read and includes numerous cartoons to help non-familiar readers understand the anatomy and development of Ciona's heart, a non-conventional model.

We thank the reviewer for their cogent summary of our work and their appreciation of the overall strength of this study.

However, the current manuscript has issues that need to be addressed:

The authors proposed that the juvenile heart has cardiac precursors dividing symmetrically after leaving the UL and before differentiating into cardiomyocytes. While it is an appealing hypothesis, the authors do not have direct evidence. They observed cell pairs in Figure 2H. These cells might divide symmetrically in the UL before leaving instead of after. Moreover, they do not seem Edu-positive. Did the authors ever observe EdU-positive cells in the myocardium of juveniles after the 30-minute EdU-pulse (lines 326-329)? The author might expect the event to be as frequent as the asymmetric midline UL division (lines 351-352).

We agree that our data in support of the presumed symmetric division of the myocardial precursors is weak and have decided to remove this hypothesis and associated observations from the current paper. We aim to more rigorously characterize myocardial precursor/myocardial proliferation in a future study, keeping this paper focused on the fate and proliferation of progenitor lineages. We feel that this paper is already very long and covers a wealth of new data (as pointed out by reviewer 2 who considers the number of figures to already be “unwieldy”) and are hesitant to add an additional section focused on myocardial precursor proliferation, particularly as this division is not central to our model regarding the role of the UL progenitor line in heart growth.

In particular the following edits were made:

We removed Figure 2H and the associated description from the results section.

Line 343 “~~Interestingly, we noted that these presumptive myocardial precursors were present as singlets just anterior to the UL (Fig 2A and 2B), while they appeared as doublets when they were located closer to the myocardial nuclei (Fig 2H).~~

Line 351 “The newly produced precursors then detach from the UL~~, migrating~~ and migrate anteriorly along existing myofibrils as they ~~divide to produce two differentiating myocardial daughters~~ differentiate.”

Why are the authors not considering the distal clusters as the most undifferentiated/stem-like cells and the midline cells as the "transit-amplifying zone" in juveniles?

We appreciate this suggestion and agree that this may be the case. However, as more explicitly stated in our revised discussion (see below), we recognize that more work is required to fully characterize distal vs. midline lineages and so we have decided to put off labeling midline cells in this way until further data is available in support of this hypothesis. Additionally, based on these considerations along with the points made above regarding the paucity of data regarding juvenile myocardial precursor proliferation we decided to also forego using the term “transit amplifying zone” in reference to the presumptive adult heart region containing myocardial precursors and instead refer to this region as a “transitional zone”. We have changed the text as below:

Line 260 - The proliferative distal UL border cells may represent a ~~transit amplifying~~ transitional zone, in which myocardial or pericardial precursors undergo proliferation prior to differentiation (as illustrated in Fig ~~1I~~1J).

Discussion, Line 934 - We ~~are currently working to identify~~ recognize that our data in support of these presumptive asymmetric divisions is limited. To more rigorously characterize distal and midline progenitor lineages along with presumptive myocardial precursors, future work will focus on identifying genetic markers to robustly distinguish between each of these populations.

Furthermore, cell division perpendicular to the UL midline with one daughter cell not anymore in contact with the anterior side of the UL is not a sign of asymmetric cell division since both MUV1B and MUV1A (Figure 2I and 2J) stay in the UL in contradiction with line 957-958 of the discussion.

This is a great point. To clarify, this line in the discussion relates to the division of MUV0 in our model, which results in the production of a midline progenitor (MUV0A) and a myocardial precursor (MUV0B) as seen in a some of our lineage tracing experiments.

We also appreciate that the distinction we are trying to make between symmetric vs. asymmetric midline UL divisions was not clearly defined. To address this issue, we have added some definitions to the relevant text (blue font below), clarifying that our data indicates that midline progenitor undergo both symmetric and asymmetric division patterns and how we distinguish between them.

Line 362 - “Despite this challenge, we were able to document ~~both symmetric (Figs 2J and S4A) and asymmetric~~ divisions of midline UL cells ~~(Figs 2J, S4B and S4C~~ that appeared to be either symmetric (Figs 2I-L and S2A) or asymmetric (Figs 2I-L, S2B and S2C) as predicted by the EdU clonal data. In these studies, we defined division symmetry based on the relative position of the resulting daughter cells. If both daughter cells remained aligned with neighboring UL cells (and in the same focal plane), we considered this as representing a symmetric division. On the other hand, if one daughter was aligned with neighboring UL cells while the other daughter was no longer aligned (having been displaced either anteriorly or onto a different Z-plane) we considered this as representing an asymmetric division.

While the single-cell data is from the adult heart, building the transcriptional trajectory from the UL to the cardiomyocytes would be interesting to see if a cell state corresponding to cardiac precursor cells is present. In this regard, mapping the different UL subclusters might also be informative.

We appreciate this suggestion. Unfortunately, we were not able to effectively generate the recommended trajectory using available data. We aim to conduct additional scRNA-seq assays using sorted juvenile heart cells as this may allow us to more effectively examine the transcriptional trajectory between progenitors and myocytes.

In addition, could the cells with a distinctive morphology in the myocardium be a different cell type, such as immune cells?

We thank the reviewer for this interesting question. Although some cells with distinctive morphologies in the myocardium could indeed be immune cells we are not currently able to identify them as such. Instead, we have focused on assessing support for our hypothesis that a subset of these cells, particularly those that are located just posterior to the UL, represent myocardial precursors as supported by our EdU+ clonal data (2G). Additionally, our hypothesis that cells in more anterior positions closer to the myocardial band also represent myocardial precursors is supported by single cell tracking data (Fig S2 D and E).

Plots displaying the data do not always match their statistical test.

In panels 4B to 4D, stacked bar charts represent qualitative/categorical data. The authors should use the Chi-squared test of independence to test the statistical difference. T-test should be used to test the statistical difference between two samples of a normally distributed quantitative variable. Quantitative data is represented by dot plots, box plots, or violin plots, showing the distribution of the samples, as shown in Figures 5E and 5J. The authors should use a Mann-Whitney U/Wilcoxon rank sum test if the data are not normally distributed. For multiple comparisons (more than two groups), the authors should first perform a One-Way ANOVA followed by a t-test with a correction for multiple testing (such as Bonferroni).

Was correction for multiple testing applied for the tests in panel 5J, S2A? Are the data normally distributed?

We thank the reviewer for their useful suggestions regarding statistical analysis. We first reanalyzed the data from Fig4B-4D and 4G using a Chi-squared test. Then to address the concerns regarding Fig5E and 5J we performed the Shapiro-Wilk test to determine if data are normally distributed. Note we also changed the quantification of Figure 5J (now 5Q) by combining both distal clusters together into one category (previously ventral and dorsal were separate). We determined that Figures 5E and 5J (now refer to 5J and 5Q) were not normally distributed. We then performed a Mann-Whitney test and determined that there was a significant difference between control and aprepitant treated animals when examining EdU counts in the distal UL cells, but not the middle UL cells (Fig 5Q). For Fig 5E (now 5J) we performed the Mann-Whitney test followed by Bonferroni correction, which revealed there was a significant difference between control and spantide-II, and control and aprepitant, but no significant difference between aprepitant and spantide-II as predicted by our hypothesis.

Moreover, if the authors have the cell quantification, it is better to display cell numbers instead of proportions.
We thank the reviewer for their suggestion, however our cell number quantification for the EdU data contains many zero values, and thus we found that it is more appropriate to represent the data as proportions rather than displaying cell numbers.

Panel 4G plot also displays a categorical variable (presence or not of more than the average + one standard deviation). A t-test should not be used.

We agree with the reviewer’s helpful comment and have reanalyzed the data accordingly.

In panel 7b, the violin plot should be for continuous quantitative data. (Stacked) bar charts show proportion. Fisher's exact test is then correct.

We also thank the reviewer for their comments in regards to panel 7b. We changed the representation of the data to a bar chart instead of the original violin plot. As there are only two categories we only included % abnormal instead of a stacked bar chart as suggested. Also as suggested we performed a Fisher’s exact test between each respective CRISPant and control, which revealed a statistically significant difference for both tachykinin and tachykinin receptor. We have now performed multiple testing using Bonferroni correction for the relevant data as suggested.

The Neural/Pacemaker (NP) subclusters are characterized in detail. However, it deserves some clarification.

We thank the reviewer for the following questions and comments regarding this dataset.

How many cells were assigned to the NP cluster and then to the different subclusters?

There are 54 cells in NP-A, 50 cells NP-B, and 26 cells NP-C as specified in the revised results section, lines 631-638.

The heatmap in panel 3I shows that NP-B is both Chat- and Th-positive. However, the authors do not observe double-positive cells with reporter assays for Th and Vacht.

We thank the reviewer for pointing this out. We have tried to clarify that this sub-clustering analysis is not definitive.

Line 628 - “To determine if there are indeed transcriptionally distinct neural-like cell-types contained within Cluster 8, we performed an additional Louvain clustering analysis on this cluster alone leading to the identification of 3 ~~sub-clusters (Fig 3G).~~preliminary sub-clusters (Fig 3G, note that these subclusters are not definitive and may each represent a number of distinct cell types with similar expression patterns).”

We also have modified our description of this data to make it clear that our analysis indicates that we can discern separate populations, instead of indicating that no double positive cells are present.

Line 669 - Using double *TH>Kaede*, *VACHT>CFP* lines, we were able to discern distinct populations of Kaede (TH+) and CFP (VACHT+) cells ~~do not overlap~~ within the ventral plexus (S5D Fig).

What is the expression level of Th, Chat on the UMAP projection of the NP cluster?

TH is expressed in many cluster 8 cells at a high level (normalized expression value of ~10). Vacht, on the other hand, is expressed in very few cells within this cluster, but in these cells, it is expressed at a high level (normalized expression value of ~10). Due to space limitations, we did not include this data or supporting images from our UMAP analysis in the paper.

Panel S9B shows Vacht- and Pc2-double-positive cells. Is the co-expression a transient developmental effect? How is Pc2 expression on the UMAP projection of the NP cluster?

This is another good question! We agree that this may be a transient developmental effect (specific to juvenile hearts) and have added some language to the paper to address this point.

Line 691 - “In summary, our reporter analysis combined with our scRNA-seq data indicates that there is a complex array of neural–like cell types in these early distal plexuses and suggests that there may be shifts in the composition of these cardiac neural-like cells as the heart matures.”

But as clarified above, we also are careful to emphasize that our sub-cluster analysis is preliminary at this point. As mentioned in the discussion, we aim to more definitively characterize different cell types represented within the cardiac “neural-like” cluster in future work. Considering the already extensive scope of the findings in this paper, we do not feel it would be appropriate to try to include additional analysis in this paper.

PC2 is highly expressed in many cluster 8 cells, normalized expression value of ~10. Due to space limitations, we did not include this data or supporting images from our UMAP analysis in the paper.

Are NP-A expressing any marker for a given neuron type, such as GABAergic or glutamatergic?

Cells in NP-A express the vesicular glutamate transporter SLC17A8, however some cells in NP-B also express this marker. Further studies with reporter or juvenile single cell data will be needed to more definitely characterize these neuronal subtypes. As mentioned above, we feel that such analysis is beyond the scope of the current paper.

The authors also argue that NP-B represents a mix of neural and pacemaker cells derived from the mesodermal lineage.

We appreciate this set of concerns regarding our hypothesis that the neural-like subcluster may contain pacemaker cells. Due to the current paucity of our data in support of this hypothesis, along with Reviewer 2’s concern that the paper is already unwieldy, we have substantially cut back our focus on this hypothesis. Critically, this hypothesis was not related to any of the key findings of the paper and thus removing much of this data/interpretation allowed us to provide a more narrow, productive focus on our three central findings (cellular mechanisms of heart growth, role of Wnt is suppressing progenitor proliferation and the role of tachykinin in promoting progenitor proliferation). The changes we have made in this regard are detailed below.

However, NP cells in Figure 3D do not express Tnni1 (Troponin) or any of the cardiomyocyte markers. What is the Tnni1 expression level on the UMAP projection of the NP cluster? Are the panels S8D showing double reporter assay for Vacht and Troponin maximum intensity projections? Where are these pacemaker cells in the cartoon in panel S8A?

We have removed the data regarding overlapping expression of TNNI and Vacht reporters (Fig. S5) and associated text.

Strikingly, in vertebrates, pacemaker cells are not neurons but specialized cardiomyocytes concentrated in specific zones. They are hyperpolarized and able to generate an action potential. These cells are innervated by cholinergic neurons, among other neuron types. Are the authors hypothesizing that pre-vertebrate cholinergic pacemaker cells (one NP-B subtype) subfunctionalizes to give rise to vertebrate pacemaker cells? Neurons in mice also expressed Hcn4. Why would the cells be cholinergic? Where are cholinergic receptors expressed in the heart UMAP?

These are excellent questions/comments and we hope to address them in a future study focused specifically on identification and characterization of cardiac pacemaker cells in *Ciona*. However, expression of Hcn4 is strongly associated with vertebrate pacemakers so we have retained our interpretation that Hcn4 expression within a subset of cells in the neural-like cluster may indicate that presence of pacemaker cells in this cluster. However, we have tried to rephrase this part of the results section to make it clear that this interpretation is preliminary.

Line 646 - “Based on this analysis, we hypothesized that NP-A represents a population of intrinsic cardiac neurons while NP-B and NP-C represent mixed populations ~~of~~ containing both intrinsic neural and pacemaker cell types. Note that this interpretation is highly provisional. As detailed below, we have conducted reporter analysis for a limited set of sub-cluster specific marker genes. However further studies will be required to accurately characterize cell types represented by this cluster.”

Moreover, the instances of Phox2b-positive mesodermal-derived cells are not convincing. Phox2b is a cytoplasmic reporter (Unc26). Nevertheless, it seems nuclear when it is co-expressed with the Mesp reporter. Might this signal be autofluorescence associated with dead cells?

We have removed the data regarding the overlap between Phox2B and Mesp reporters.

Since the adult heart is still growing, could the NP-C subcluster represent a precursor state of NP-B?

Although this is an interesting suggestion, we do not currently have enough data to evaluate hypotheses regarding precursor states within presumptive cardiac neural-like clusters.

Based on the fluorescence in situ hybridization (Figure S12), it is possible that Tacr is more broadly expressed at the juvenile stage than in the adult. Neuropeptides usually rely on volume transmission and not on precise synaptic transmission. They might diffuse more than 100µm from their source. It might explain the dramatic phenotype observed upon Tacr CRISPR using Cas9 driven by Mesp regulatory sequences, matching the phenotype observed in Tk CRISPR juvenile. Tk would then directly provide pro-proliferative signals to UL.

We appreciate this intriguing suggestion. However, the in situ data (now shown in Fig. S8) does not indicate that TACR is expressed in the UL progenitor lineage of juvenile hearts. As we more explicitly emphasize in the revised discussion the juvenile TACR in situ data is not very clear and further studies will be required to assess which cell types respond to TK.

Line 1000 “One major gap relates to the specific identity of cells that respond to tachykinin signaling. To overcome this gap, it will be critical to perform a more robust characterization of *TACR* expression in adult and juvenile hearts, as our current data do not clearly delineate which cell-types express *TACR*, when this expression pattern emerges or whether this pattern shifts during heart growth.”

Additional comments:

* The number of analyzed samples is not always mentioned.

corrected

* Line 568, 588, 620, 670, 684, 786, 788, 856 1012 reference the wrong figure.

corrected

* Scale bars are missing in panels 2A, 2J, Figure 5, panels 7A, 7E, 7F, S1A, S1B, S1C, S1D, S1E, S8B, S8C, S8E, S8F, S8D'' (named S8D'), Figure S10, panel S11B, S11G, S11H, Figure S13

corrected

* In the S1A legend, the authors mentioned XY projection. It is an XZ projection in the figure.

This figure panel was removed.

* Gene names are not always consistent; for example, Gata4/5/6 (line 457) instead of Gata.a and CrUL1 in the main text is named UL1 in Table S1 and Panel 7I and 7J or UL-1 in Figure 3.

Corrected. However, in the cited case of GATA (now line 454) we used Gata4/5/6 to indicate the vertebrate orthologs while we used Gata.a as this is the currently annotated *C. robusta* gene name.

* Gene and plasmid formats or names are not uniform. For example, the format of gene names in Figure 3 or Mesp is in uppercase, while Phox2b is capitalized (line 1856 and Figure S10). Some fusion proteins have one ":" others two (line 1856 and Figure S10).

Corrected, for this specific example the figure panel was removed.

* Wnt signaling might promote differentiation. In this case, its inhibition blocks differentiation. The cells stay as progenitors and keep proliferating. The effect of Wnt on proliferation at this stage might be indirect.

We appreciate this suggestion and have included it in our revised discussion.

Line 966 - “Further studies will also focus on determining whether cWnt signaling acts directly to drive progenitors into a mitotic arrest or if it acts indirectly, for instance by promoting myocardial differentiation of progenitor daughter cells or by fate specification into another less proliferative cell type.”

* Neural-like cells could be referenced as neural cells.

We prefer to use neural-like cells to encompass the potential representation of pacemaker cells in this cluster.

* Annotate the heart outline in Figures S8B and S8C.

Corrected

* Phox2b is enriched in both NP-B and NP-C, not NP-A (Line 699)

This data was removed.

* The drug Spantide II is referenced as Spantide I in the legend of Figure 5 and Spantide in the figure.

Corrected.

* Some references miss the journal name, or their abbreviation is not consistent.

Corrected

In summary, this manuscript represents critical steps into our comprehension of heart development in Ciona juveniles, which will enhance our understanding of the origin of vertebrate cardiac innovation, including its intrinsic innervation. I support its publication in PloS Biology, contingent upon the recommended modifications.

We once again thank this reviewer for their thoughtful critique and strong support of our study.

Reviewer #2: In this manuscript the authors use the chordate model Ciona robusta to investigate post larval heart development. Specifically they are interested in neuronal signals that regulate heart growth. The effects on cell proliferation and heart growth are examined after manipulation of the canonical Wnt pathway using pharmacological tools, or CRISPR to inhibit tachykinin neuropeptide and tachykinin receptors. They propose that insights gained may be useful for understanding vertebrate heart growth.

I appreciate the amount of work that has gone into this manuscript. However, I found the paper unwieldy, the number of supplementary figures (13) and figures (7) is unbalanced and the different parts could be better integrated.

We thank the reviewer for this comment. To better balance the number of supplementary vs. main figures we have integrated key data from S1 and S2 into Figure 1 and combined three other pairs of supplementary figures (S6 with S7, S9 with S10 and S12 with S13) leaving us with a total of 8 supplementary figures. As detailed in response to Reviewer 1, we have also removed some excess supplementary data related to derivative lines of inquiry that were not directly relevant to our main findings.

The introduction is focused on mammals and some other vertebrates (which are often not identified for the reader).

We thank the reviewer for identifying these ambiguities in our introduction. We have addressed this concern by explicitly noting in our initial introductory paragraph when we are referring to studies from a range of model organisms or specifically to vertebrate studies.

Line 52 - “Studies in a wide range of organisms have shown that neural signals regulate development and patterning of both neural and non-neural cell lineages (1-3).”

Line 57 - “Initial studies revealed a role for innervation in vertebrate skeletal muscle differentiation”

Line 61 - “Additional research in both vertebrate and invertebrate model organisms has extended the range of morphogenetic processes…”

Overall, this introduction does not link well with the subject being studied, and it is also not picked up in the discussion.

We respectfully disagree with this reviewer's critique of the introductory section of this paper. The introduction begins with a broad overview regarding the role of neurons in development and gradually narrows to focus on other relevant topics including an overview of vertebrate heart growth, signals that regulate vertebrate heart growth, neural signals that regulate this process, the use of Ciona to study neural inputs on heart growth, what we know about the heart and cardiac innervation in Ciona along with a brief summary of our findings. For the middle section of the introduction, we focused on vertebrate heart growth because the majority of findings regarding this process have come from vertebrate studies and because insights from Ciona and vertebrate models are likely to inform each other due to possible conservation of these mechanisms within the chordates. However, in response to this reviewer’s comment we have added a citation to a recent review regarding studies of heart development in *Drosophila* and modified the beginning of the relevant section to read -

Line 84 - “Signals that regulate ~~vertebrate~~ heart growth…”

Line 85 - “Extensive research in both vertebrates and fruit flies has documented signal-dependent coordination of initial cardiogenic patterning along with homeostasis and regeneration of the mature organ (46–55).”

As the other two reviewers did not express this concern, we hope that we are able to address this reviewer’s concerns by making these edits instead of changing the overall format or flow of the introduction.

Much of the discussion is focused on what they aim to do next rather than trying to make sense of their observations.

We appreciate this reviewer’s concern but feel that a focus on follow up studies is appropriate for this paper. Our findings represent a new frontier both in regards to investigating heart growth in *Ciona* and in probing the role of tachykinin in heart growth in any organism. Although we have made substantial initial headway on both of these fronts our findings raise many more questions than they answer.

Additionally, the initial paragraph of the discussion constitutes an overview of our findings and in each subsequent paragraph we touch on our current understanding before touching on the questions that are raised by these findings and how we intend to address them. Thus, we feel that we do dedicate a substantial part of the introduction towards making sense of our findings.

The first two figures describe the microscopic anatomy of the adult C. robusta heart and of the juvenile heart. While this would be of interest for a specialist reader, it is difficult to follow the evidence supporting the cartoon overview models shown. These cartoon models are Fig 1I for a presumptive transit amplifying zone; and Fig 2I for the undifferentiated line (UL) and myocardial precursor cell divisions. The challenge is the very low proliferative index and UL cell divisions were rarely observed. The core premise is therefore not convincing, line 387: "our data strongly supports the following core premise:..."

As mentioned in response to the concerns raised by Reviewer 1, we no longer posit a transit amplifying zone. Instead, we simply refer to this area as a “transitional zone” in both the figure and the results section. This is a more straightforward interpretation of our current data and will hopefully be more useful to our readers.

In regards to the diagram in Figure 2 (now 2H) we feel that this schematic is critical for summarizing the data we obtained through cell lineage tracing and in illustrating our resulting model of progenitor proliferation within the UL.

Although it is true that UL cell divisions were rarely observed, we were able to overcome this challenge in two ways. First, we were able to analyze numerous EdU+ pulse-chase assays. As clarified in the revised results section, we were able to rigorously document that a 30-minute pulse only labeled one proliferative UL cell, allowing us to assess resulting clonal patterns after a 72-hour chase in 14 juvenile heart samples in which we found a clone containing at least one EdU+ UL.

Line 339 - “We then assessed the composition of presumed clones across 14 independent samples that contained at least one EdU+ UL cell.”

The clonal patterns that we observed using this approach are documented in Figure 2 D-G. Additionally, through extensive photoconversion analysis of Kaede-labeled UL cells, we were able to further document a number of key division patterns (Fig. 2I-L and Fig S2) which are represented in this schematic.

We also wish to point out that all we are claiming is that our data supports the premise that the UL serves as a progenitor lineage for myocardial cells, not that this is a definitive conclusion. Indeed, we explicitly state our caution in relaying this interpretation, stating that “**Although the details of this model may shift as more data is collected**, our data supports the following premise: The UL constitutes a cardiac progenitor population that plays a central role in myocardial growth. In accordance with this premise, we will subsequently refer to UL cells as cardiomyocyte progenitors.”

To ensure that we do not state this conclusion too strongly in the discussion we have revised relevant statements as follows -

Line 907 - “We first ~~demonstrated~~ provide lineage tracing data indicating that the undifferentiated line serves as a stem-like progenitor population capable of producing new myocardial cells (Fig 2).”

Line 929 - ~~“According to our current model of C. robusta heart growth~~ Our data suggests that new myocardial cells are derived from three distinct populations - distal and midline progenitors that reside in the undifferentiated line along with myocardial precursors that migrate anteriorly before differentiating.”

line 396, there is no citation for the protocol from the Klein lab; citation number [87] is incomplete

We thank the reviewer for pointing this out. It was an error made when formatting the bibliography. We have made the appropriate change and now reference the Klein Lab’s work accurately.

The transcriptomic analysis provides several clusters of cells for two stages of adult hearts. They focus on a cluster (no. 7) representing cardiomyocyte progenitors of the UL and validate expression of one selected gene, CrUL1, by in situ, S7 Fig. The pattern observed was however different to that of their reference gene, Gata.a. This is confusing and was not commented on any further.

We thank the reviewer for bringing this up. As they note, the expression pattern we observed for *Gata.a* in the juvenile heart encompasses both the UL and myocardial cells, while our scRNA-seq data indicates that *Gata.a* is highly enriched in the UL. As pointed out in our revised assessment of this result, this discrepancy may reflect a shift in expression associated with heart maturation. We have amended the text to clarify this point.

Line 484 - “As predicted by our adult heart scRNA-seq data, *Gata.a* is highly expressed in the UL ~~along with myocardial cells~~ in juvenile samples. However, we also observe *Gata.a* expression in the juvenile myocardial cells. The apparent loss of *Gata.a* expression in the presumed adult myocyte sc-RNA-seq cluster (Fig 3B) may reflect definitive myocyte differentiation during the juvenile to adult transition.”

The data in Figure 4 shows clearly that pharmacological inhibition of the canonical Wnt pathway leads to an increase in the frequency of cardiomyocyte progenitor division. This is interesting and unexpected. They next show that pharmacological inhibition of tachykinin peptide signalling reduces the number of progenitors. This is somewhat supported by knockdown of tachykinin or its receptor using CRISPR-Cas9 approaches, which are mosaic and lead to a variable phenotype with partial penetrance. In some cases the formation of the myocardial tube is affected.

As mentioned in relation to this data, the partial penetrance that we observe using this technique is consistent with what has been observed in previous studies when CRISPR-Cas9 is deployed in Ciona. As has been detailed in these earlier studies, we use transient transgenic assays to introduce Cas9 and sgRNA constructs and the level of incorporation of these constructs can be highly variable across different embryos or juveniles. Additionally, these constructs are often incorporated mosaically, leading to further variation in the resulting crispant phenotypes. Furthermore, extensive genetic polymorphism within C. robusta populations can further reduce the incidence of successful mutagenesis. We have cited a number of additional references that have previously delved into these various issues.

“The partial penetrance of this phenotype aligns well with other CRISPR-Cas9 assays in *C. robusta* and is considered to reflect mosaic incorporation of plasmids (148-151)”

We have further buttressed our support for this key finding in two ways -

1. We have conducted an additional pharmacological manipulation involving treatment of juveniles with Ramipril, an inhibitor of a key enzymatic pathway that degrades tachykinin. As predicted by our hypothesis, this gain of function assay resulted in a substantial and significant increase in progenitor numbers. This new experiment is described in the results section as follows and illustrated in Fig. 5J-L.

“We also performed a gain of function experiment, treating D3 juveniles with Ramipril, an inhibitor of the angiotensin-converting enzyme pathway that breaks down tachykinin (143). As predicted by our hypothesis, treatment with Ramipril resulted in a robust and significant increase in the number of midline progenitors in comparison to controls (Fig 5J-L).”

2. We have rigorously analyzed CRISPR mutagenesis of TACR using the same sgRNAs as we deployed for this study. We also attempted to perform this analysis for the TK sgRNAs but the targeted locus was flanked by A/T rich regions that prevented amplification of this locus as required for further analysis. This new data is summarized in revised Figure S8 and mentioned in our results section as documented below.

Revised results section –

Line 859 –As observed in the *TK* knockdown assay, loss of *TACR* severely disrupted heart morphology, generating a similar hollow pericardial cavity phenotype (Fig 7B, E and F, S3-S4 Movies). As mentioned in relation to *TK* knockdown, partial penetrance of this phenotype likely reflect mosaic incorporation of plasmids [149]. Additionally, partial penetrance may result from population-specific sequence variation in the targeted region (S8 Fig, [152]).

Revised methods section –

Line 1262 - After experiments were conducted, the targeting efficiency of deployed sgRNAs were tested in larvae co-electroporated with *Ef1a>Cas9* along with either *TACRsgRNA1* or *TACRsgRNA2* and then subjected to Azenta Genewiz SNP/Indel analysis as described in [152]. Genomic DNA was harvested from a large pool of transgenic larvae (~500) and the region targeted by each sgRNA was then amplified by PCR and sent in for sequencing. For each sample, ~1000-1500 unique reads were analyzed for the presence of naturally occurring IN/DELs and for mutagenesis within the targeted locus. This analysis revealed a highly prevalent naturally occurring IN/DEL near both targeted loci. In these reads, there was a high level of sequence variation in the targeted locus. Thus, we manually filtered through the unique reads generated by this analysis for each sgRNA (*TACRsgRNA1* and *TACRsgRNA2*) to distinguish whether or not they contained the prevalent IN/DEL. We then assessed the incidence of mutagenesis within the unique reads that did not contain the IN/DEL. See the S8 Fig legend for additional details.

Revised legend for Figure S8 –

Line 1431 - **(E)** Table displaying data generated from transgenic larvae co-electroporated with *Ef1a>Cas9* along with either *TACRsgRNA1* or *TACRsgRNA2* and then subjected to Genewiz SNP/Indel analysis. For each sample, ~1000-1500 unique reads were analyzed for the presence of naturally occurring IN/DELs and for mutagenesis within the targeted locus. This analysis revealed a highly prevalent naturally occurring IN/DEL near both targeted loci (67% for *TACRsgRNA1* and 91% for *TACRsgRNA2*, column three). In these reads, there was a high level of sequence variation in the targeted locus (Supplementary Table 3). Thus, we manually filtered through the unique reads generated by this analysis for each sgRNA (*TACRsgRNA1* and *TACRsgRNA2*) to distinguish whether or not they contained the prevalent IN/DEL. We then assessed the incidence of mutagenesis within the unique reads that did not contain the IN/DEL (17% for *TACRsgRNA1* and 18% for *TACRsgRNA2*, column six). These percentages align with the penetrance observed when both sgRNAs were used for CRISPR (Fig 7). We suspect that the high incidence of the IN/DEL may reflect a change in the *C. robusta* population used for this follow up assay. This sequencing assay was conducted using gametes from adults harvested from a Los Angeles population that is distinct from population used in our CRISPR experiments. As expected for targeted mutagenesis, all detected deletions that potentially resulted in a frame shift occurred within 60 base pairs of the protospacer-adjacent motif (PAM) with a peak that occurred 3 base pairs upstream of the PAM (Supplementary Table 3).

The relationship between canonical Wnt and tachykinin pathways is not clear and not explored further and discussion is very limited to: line 1017 "We are also interested in investigating whether TK signaling instructs neural-like cells to secrete cWnt inhibitors."

We appreciate this comment and are very interested in the potential intersection between these two signals. We have added an additional sentence to the discussion to clarify the speculative hypothesis that we put forward.

“Line 1013 - We are also interested in investigating whether TK signaling instructs neural-like cells to secrete cWnt inhibitors, thereby blocking Wnt-dependent suppression of progenitor proliferation (Fig 4) in order to promote heart growth.”

Although we agree that it would be interesting to speculate further regarding potential intersections between our data on the roles of TK vs. Wnt in this study, our current data does not provide a link between these two, potentially distinct, inputs on heart growth. As mentioned above, we agree with this reviewer’s assessment that the paper is already somewhat unwieldly due to the amount of data that we are trying to summarize and present. Thus, we strongly feel that further experimental analysis regarding potential interactions between Wnt and TK signaling is beyond the scope of this study and that it will be more appropriate to provide a more in-depth discussion of these interactions in a follow up paper that is focused on this line of inquiry.

Given that the introduction is focused on mammals, the authors should consider how their findings are relevant to vertebrates. Alternatively, change the focus of the introduction.

We agree that it is important to consider the relevance of our findings in relation to vertebrate cardiogenesis. As pointed out by this reviewer, our discussion of this subject is limited and we only mention it a couple of times in the discussion (see excerpts below). Instead, much of our discussion is focused on the current limitations of our data and the need for future studies to enhance our understanding of Ciona heart innervation and growth along with the specific impacts of Wnt or TK on cardiomyocyte progenitor proliferation. Considering that our study is the first foray into these topics, we think this is the appropriate focus for the discussion and strongly feel that it will be more productive to further discuss the relevance of our findings in regards to vertebrate cardiogenesis once we have consolidated these findings through the proposed follow up studies.

Line 972 - A more coherent understanding regarding the role of cWnt signaling in *C. robusta* heart growth will allow productive comparisons with vertebrate cardiogenesis, potentially delineating specific functional similarities. One of the most exciting results of this part of our study was that inhibition of cWnt signaling led to an overall increase in heart size without apparently impacting heart morphology. Thus, we anticipate that insights into the role of cWnt signals in *C. robusta* heart growth will inform a broader understanding of proportional organ growth.

Line 1024 - Though many studies have revealed roles for innervation in promoting organ development, few studies focus on the impact of innervation on progenitor or stem cell proliferation [12, 13, 16]. Literature on potential roles for cardiac innervation or neuropeptides in modulating cardiomyocyte progenitor proliferation generally focus on post-natal growth or injury [23, 81, 141, 159]. Thus, insights gained from further exploration of heart formation and growth in *C. robusta* may inform efforts to better understand the role of tachykinin and other neural signals in vertebrate heart development.

Other issues:

line 684, "these distinct neurons may be represented by sub-clusters NP-A or NP-B respectively (Fig 2I)." It is not clear why the authors refer to this cartoon model here.

We thank the reviewer for noticing this. This line has been removed during revision.

line 720-722, "Application of the TH inhibitor α-methyl-para-tyrosine (AMPT) lowered heart rate slightly, but this result was not significant. In contrast, application of the acetylcholine agonist acetylcholine chloride and the TACR inhibitor aprepitant had no discernible impact on heart rate." This does not make sense. Why do the authors say "in contrast" when in both cases there was no effect.

We thank the reviewer for noticing this typo. We agree, and have replaced the phrase “In contrast” with “Furthermore” in this statement.

line 939, "we posit that both distal and midline progenitors behave like cardiac stem cells, dividing asymmetrically to self-renew while producing a midline or precursor daughter respectively (Fig 2I). We are currently working to identify genetic markers that distinguish between these two progenitor populations." Again as above, I am not convinced that there is sufficient evidence to support this.

We thank the reviewer for this comment. We agree that this hypothesis is not definitively supported by our data. However, we do feel that we have enough data (as discussed in response to a similar concern raised by this reviewer above) to support our model and have made it clear in our revised discussion that this model requires additional testing, as explicitly mentioned in the revised section of the discussion cited below.

Discussion, Line 933 - We ~~are currently working to identify~~ recognize that our data in support of these presumptive asymmetric divisions is limited. To more rigorously characterize distal and midline progenitor lineages along with presumptive myocardial precursors, future work will focus on identifying genetic markers to robustly distinguish between each of these populations.

Reviewer #3: Review of the manuscript by Gruner et al.

The paper by Gruner et al. provides novel insights into how neural signals are involved in the development of heart cells during Ciona robusta development. The manuscript is well written, and the experiments are comprehensive and clearly described. The results are thoroughly discussed and are of significant interest to the community.

The authors begin with a detailed analysis of the cellular composition of the heart in both juvenile and adult Ciona, with a particular focus on the undifferentiated lineage (UL). The proliferative potential of different cell populations was assessed using EdU pulse-chase experiments, revealing a specific pattern of mitotic activity in both juveniles and adults. Specific asymmetric division in juvenile UL supports the conclusion that the UL represents a source of undifferentiated, proliferative cells.

Single-cell transcriptomic analysis of isolated heart tissue identified distinct subpopulations of heart cells. Notably, the authors identified a population of cells putatively associated with neural-like cell types through scRNA-seq analysis. Using stable transgenic lines and a photoconvertible marker (Kaede), they were able to trace, characterize, and investigate the potential role of these cells during heart formation.

The authors make appropriate use of a wide range of experimental approaches, allowing them to address the scientific question from multiple perspectives. Functional studies using CRISPR/Cas9 gene editing and pharmacological inhibitors revealed key roles for Wnt signaling and tachykinin in regulating the growth and differentiation of heart cells.

Overall, this study represents an important contribution to the field highlighting *Ciona robusta* as a powerful model for studying the interplay between neural signals and cardiac development.

We thank the reviewer for this cogent and positive summary and review of our work.

Second Response to Reviewers

We thank the reviewers for providing further feedback on our manuscript and have addressed their remaining concerns as detailed below.

Reviewer #1
- In the caption of Figure 4 (lines 588-589), p-values are mentioned as asterisks. In the figure, they are in numbers. Corrected.
- What statistical test was done for Figure 4G? I could not find it in the caption or the method. I still think that it would be easier to understand a (violin/dot) plot with the UL cell number for this experiment if the authors have the exact cell number. Addressed by adding an explanation to the methods section.
- In lines 726 and 727, the author mentioned to panel 5E and not 5D. Corrected
- In the caption of Figure 5P (line 760) and the statistic section of the methods, quantification of EdU+ cells is divided into dorsal, ventral, and midline UL, while the plot now shows distal and midline UL. Corrected
- The statistical tests in Figure 5I, 5L, 5P are Mann-Whitney in the caption, but t test in the statistics section of the methods. Corrected
- In the statistics section of the methods (line 1071), the adult heart is divided into distal, middle, apical. In Figure 1K, it is divided into distal and middle. Corrected
- The caption of Figure 7B (line 881) mentions a violin plot, while it is a bar plot in the figure. Corrected


Reviewer #2
Some of the panels in figure 7 contain (?) (7D, F, H, J). I could not see where this is explained in the text or the legend. Sorry - I missed this before. Addressed by adding an explanation to the figure legend.

The references are not always correctly formatted, e.g. refs 1,3,5, 12,14, 15, 23 have no Journal, ref 48 has no title Corrected
